# Strengthening Emergency Care Systems to Mitigate Public Health Challenges Arising from Influxes of Individuals with Different Socio-Cultural Backgrounds to a Level One Emergency Center in South East Europe

**DOI:** 10.3390/ijerph15030501

**Published:** 2018-03-12

**Authors:** Michèle Twomey, Ana Šijački, Gert Krummrey, Tyson Welzel, Aristomenis K. Exadaktylos, Marko Ercegovac

**Affiliations:** 1 Centre of Excellence in Emergency Medicine, Cape Town 7700, South Africa; twelzel@earthling.net (T.W.); aristomenis.exadaktylos@insel.ch (A.K.E.); 2 Department of Emergency Medicine, Clinical Centre of Serbia, 11000 Belgrade, Serbia; asijacki@gmail.com (A.Š.); ercegovacmarko@gmail.com (M.E.); 3 Department of Emergency Medicine, Inselspital, University Hospital Bern, 3010 Bern, Switzerland; Gert.Krummrey@insel.ch

**Keywords:** emergency care, triage, healthcare system strengthening, migrant health

## Abstract

Emergency center visits are mostly unscheduled, undifferentiated, and unpredictable. A standardized triage process is an opportunity to obtain real-time data that paints a picture of the variation in acuity found in emergency centers. This is particularly pertinent as the influx of people seeking asylum or in transit mostly present with emergency care needs or first seek help at an emergency center. Triage not only reduces the risk of missing or losing a patient that may be deteriorating in the waiting room but also enables a time-critical response in the emergency care service provision. As part of a joint emergency care system strengthening and patient safety initiative, the Serbian Ministry of Health in collaboration with the Centre of Excellence in Emergency Medicine (CEEM) introduced a standardized triage process at the Clinical Centre of Serbia (CCS). This paper describes four crucial stages that were considered for the integration of a standardized triage process into acute care pathways.

## 1. Introduction

“Emergencies occur everywhere, and each day they consume resources regardless of whether there are systems capable of achieving good outcomes” [1].

Emergency care is a critical part of a country’s healthcare system and is transversal in nature moving across different levels of care, from a community bystander response or primary care mobile clinic to tertiary specialized interventions. In a healthcare system that has fully embraced and integrated emergency care as an essential component, emergency care is everyone’s business. Health practitioners at all levels including doctors, nurses, paramedics, and first responders attend to emergencies (i.e., undifferentiated, unscheduled patients) not in the order in which they arrive but in order of acuity. Acuity goes beyond severity of illness/injury in that it includes the urgency for intervention that potentially leads to stabilization or improvement. Triage refers to the standardized prioritization process that determines a patient’s acuity [1].

In contexts where the demand exceeds the capacity to match that demand the process of triage, if appropriately developed and comprehensively integrated into acute care pathways, has shown to utilize resources more efficiently and predict mortality [2]. The World Health Organization (WHO) in a systematic review of 59 low- and middle- income countries emphasizes the ongoing need to strengthen triage as a crucial requirement for efficient resource allocation and effective emergency intervention [3]. In upper middle-income countries such as Serbia, Bosnia, Herzegovina, and the former Yugoslav Republic of Macedonia (FYROM), the demand for emergency care has been growing, and the need for continuous strengthening of emergency care systems has been recognized [4,5,6]. The Clinical Centre of Serbia (CCS) located in Belgrade is a 3500-bed medical university center that serves the population of Belgrade and larger Serbia. It is considered the largest hospital complex in Europe treating more than 1 million patients every year with a 308-bed emergency center that was established in December 1987 [7]. More than 20,000 patients enter the emergency center on a monthly basis requiring time-critical initial assessment, investigation, and intervention, including resuscitation and stabilization.

In June and August 2015, the Ministry of Health of Serbia and the WHO Regional Office for Europe conducted a joint assessment of the preparedness and capacity of the Serbian health system to manage sudden large influxes of people seeking asylum or who were in transit. The report of this joint assessment highlights the need to systematically develop both local and national policies to include migrant health needs in all levels of health planning [8]. Another independent assessment of the needs of young refugees arriving in Europe identified that physical health issues predominate upon arrival and pose significant challenges to the national healthcare system [9]. While asylum centers are set up to provide primary care and limited emergency medical procedures, the majority of people are not resident in these centers and thus mostly enter the Serbian healthcare system at a tertiary acute care level through the emergency center [8]. As such, a standardized prioritization process based on a combination of clinical discriminators and a composite early warning score was determined as relevant and appropriate for a varied acuity distribution from emergent to non-urgent, thus covering a wide variety of conditions by not being specific to only certain conditions.

Emergency medicine as a specialty is becoming more established in Serbia and emergency care has been recognized as a critical component in a transversal approach to improving population health. From September 2016 to August 2017, as part of a larger emergency care improvement and patient safety effort, the Serbian Ministry of Health in collaboration with CEEM introduced a standardized triage process at the CCS. This paper outlines four stages that were considered crucial for the integration of a standardized triage process into acute care pathways and is intended as a high level commentary to inform policy makers.

## 2. Standardize Information Gathered, Make Data Visible, and Use It to Drive Decision-Making

By gathering baseline data and making it visible, the CCS’s leadership team gained clarity on demand patterns, caseload, and current acute care pathways through the emergency center. Data collected via the hospital’s electronic patient record system (InfoMedis) was also taken into account. Initial challenges that emerged during this stage related to how the current system was set up to capture data. To obtain a baseline snapshot, a core data team consisting of four nurses and two doctors was responsible for gathering information prospectively. This allowed for the inclusion of relevant indicators relating to real-time tracking of acuity distribution of patients on or shortly after arrival, as well as value-adding information during their acute care pathway. The indicators included mode and time of arrival, age of patient, presenting complaint, vital signs, special investigations, diagnosis, treatment, and disposition. In addition, an overview of available capacity was established (number and level of skilled human resources, spaces, structures, and equipment). All information available on demand and capacity was displayed visually. This was not only essential to inform future decision-making regarding healthcare organization but also to determine how to configure and standardize a triage process that is contextually appropriate for integration into the acute care pathways. A panel of four content experts reviewed the prospectively gathered data and annual statistics from InfoMedis during three discussion rounds after which a consensus was reached on the most prevalent clinical discriminators to be included. This process informed the modifications of the triage tool for use within the local context.

## 3. Contextually Configure and Standardize the Triage Process

The literature describes many different triage tools, models, and interpretations for the emergency center setting. Some of the triage scales that are used globally include the Australasian Triage Scale (ATS) [10], the Canadian Emergency Department Triage and Acuity Scale (CTAS) [11], the Emergency Severity Index (ESI) [12], and the South African Triage Scale (SATS) [13].

The details of how the triage process manifests itself depend on the context. The most useful aspects for distinguishing different contexts for this purpose are demand and capacity, which in turn determine (i) the choice of most appropriate triage tool; (ii) the training, experience, and level of staff required; (iii) how other processes are linked to triage; and (iv) the need for task-shifting and parallel processing [14].

Most triage tools are based on a list of clinical discriminators; some include individual vital signs, while others include early warning scores (EWSs) or symptom-based algorithms. Individual vital signs considered in isolation from each other are known to be poor predictors of life-threatening conditions in patients. EWSs are known for their ability to detect physiological changes relating to vital signs [15]. Combining various standardized physiological parameters into a composite EWS has been recognized as a powerful tool in initiating appropriate responses from the initial contact at triage [16]. The benefits of an EWS include its objectivity and the fact that an aggregated score is a stronger predictor than individual vital signs and reliance on routinely recorded vital signs [17].

The CCS’s leadership team reviewed available triage instruments and chose to configure their triage process based on a modified version of SATS for three reasons: (i) comprehensiveness and safety in combining clinical discriminators and an EWS [18,19]; (ii) clarity and ease of use, which enables standardized training and reliable use [20]; (iii) evidence of widespread global adaptation and adoption from low- and middle- income countries such as Ghana [21] and Botswana [22] to high- income countries such as Norway and parts of Sweden [23]. A core training team of six doctors and three nurses was formed once a consensual adaptation process had been completed. All training material was made available in Serbian and an open-source mobile android decision support application was developed to aid the standardized training modules as well as the actual triage process for future routine use. Thus far, 30 nurse technicians have been trained in the standardized triage process, and further training is planned to cover the rest of the staff at the CCS.

## 4. Reorganize and Restructure Available Resources

A standardized triage process should ideally take place on arrival or within minutes of arrival at the emergency center in order to prioritize and stream patients into the appropriate care pathways. To introduce, integrate, and improve a standardized triage process, the necessary staff with appropriate experience and level of training, equipment, space, and decision support tools are vital prerequisites. The respective care pathways that follow the triage process are organized based on the available resources (i.e., training, experience and level of staff available as well as infrastructure, medical equipment, and supplies available) [10].

Adequate space for triage on arrival at the CCS was limited. After permission was granted for minor renovations, previously unused storage space became available and the triage station was doubled to accommodate two workstations with desk and chairs, eight stretcher patients, and eight seated patients. There are currently no emergency-medicine-trained physicians at the CCS that would be able to assist in the initial stabilization, work-up, and transfer of patients to appropriate definitive care. Therefore, the CCS’s leadership team motivated and made urgent requests for eight additional doctors with Basic Life Support (BLS) or Advanced Trauma Life Support (ATLS) training. Thus far, two doctors with some BLS training have been appointed.

## 5. Redesigning Care Pathways to Integrate Standardized Triage

Currently nurse technicians are responsible for sorting patients on arrival. Previously, patients were sent to one of four specialist-assessment areas based on the presenting complaint (i.e., neurology, internal medicine, surgery, or cardiology). The specialist assessment was performed in order of arrival and not based on the patient’s acuity. During busy periods, patients experienced long waiting times in the corridor before being seen by a specialist for assessment. This presented an increased risk for patients that were deteriorating rapidly and could not safely wait.

The target condition in redesigned care pathways is to fully integrate a standardized triage process on arrival where triage-trained nurse technicians document the crucial information gathered using a mobile android triage application. Further developments are required to link the mobile triage application to the electronic patient record system (InfoMedis). This information from the triage then determines the patient’s acuity and enables the nurse technician to decide whether the patient needs to receive time-critical intervention or can safely wait to be seen. Dangerous situations of very sick patients deteriorating, while waiting for a specialist assessment are thus kept to a minimum. The standardized process of triage and its crucial link to other functions and care pathways is described in a local triage protocol and policy document. Both guiding and supporting documents are endorsed by the Head of Department, the triage team, and the Minister of Health and intended for regular review in the future.

## 6. Conclusions

All improvement initiatives take time to reach full adoption and integration. The amount of time needed and the extent to which integration occurs depends on the political will, the resources invested, the collective leadership, and the organizational learning culture. This emergency care improvement initiative is the start of a continuous quality improvement and patient safety approach that requires on-going review and numerous iterations over time.

While the collection and collation of some indicators may not yet be routine daily practice at the CCS, the target condition is to achieve real-time data collection to allow data-driven decision-making, time-critical response, and adjustment of emergency care services for all types of unpredictable fluctuations in caseload, whether they are related to an influx in health needs of migrants or to other situational or contextual changes. The initiation of a standardized triage process is an opportunity to move toward this target while simultaneously reducing the risk of missing or losing patients with life-threatening conditions in emergency waiting rooms.
